# Intestinal fibrosis in aganglionic segment of Hirschsprung's disease revealed by single‐cell RNA sequencing

**DOI:** 10.1002/ctm2.1193

**Published:** 2023-02-03

**Authors:** Shiwei He, Junfeng Wang, Yanlei Huang, Fanyang Kong, Ran Yang, Yong Zhan, Zifeng Li, Chunjing Ye, Lingdu Meng, Yankang Ren, Ying Zhou, Gong Chen, Zhen Shen, Song Sun, Shan Zheng, Rui Dong

**Affiliations:** ^1^ Department of Pediatric Surgery Shanghai Key Laboratory of Birth Defect Children's Hospital of Fudan University Ministry of Health Shanghai China

**Keywords:** extracellular matrix, Hirschsprung, intestinal fibrosis, single‐cell transcriptomics

## Abstract

**Background:**

Hirschsprung's disease (HSCR) is a relatively common congenital disability. Accumulating extracellular matrix (ECM) prompts intestinal fibrosis remodelling in the aganglionic segments of HSCR. The contributions of various cellular subsets in the fibrogenesis of HSCR segments are poorly understood.

**Methods:**

Single‐cell transcriptomics from 8 aganglionic segments and 5 normal segments of 7 HSCR subjects and 26 healthy segments of seven healthy donors were analysed. Fibrotic phenotype and alterations were explored using differential expression analysis and single‐cell trajectory analysis. Fibrosis‐related transcription factors were inferred through single‐cell regulatory network inference. Bulk transcriptomic data, proteomic data, immunohistochemistry (IHC) and real‐time polymerase chain reaction were used to validate the alterations in the HSCR intestine.

**Results:**

Various collagen, fibronectin and laminin protein‐coding genes expression were up‐regulated in the stromal and glial cells of the HSCR intestine. The number of fibroblasts and myofibroblasts in the aganglionic segments increased, and more myofibroblasts were activated at an earlier stage in HSCR segments, which infers that there is an intestinal fibrosis phenotype in HSCR segments. The fibrotic regulators *POSTN*, *ANXA1* and *HSP70* were highly expressed in the ECM‐related cellular subsets in the transitional segments and aganglionic segments. The transcription factor regulatory network revealed that fibrosis‐related and megacolon‐related *NR2F1* in the fibroblasts and glial subsets was up‐regulated in the aganglionic segment.

**Conclusions:**

This work identifies intestinal fibrosis and related regulators in aganglionic segments of HSCR; hence, anti‐fibrotic therapy may be considered to prevent HSCR‐associated enterocolitis (HAEC), relieve intestinal stricture and improve cell therapy.

## Graphical abstract

Single‐cell transcriptomics from Hirschsprung's disease (HSCR) subjects and healthy donors’ intestines were analysed. Various extracellular matrix and fibrotic regulators of protein‐coding genes expression were up‐regulated, and more fibroblasts and myofibroblasts were activated to elucidate the intestinal fibrosis phenotype in aganglionic segments of HSCR. Anti‐fibrotic therapy may be considered to prevent HAEC, relieve intestinal stricture and improve cell therapy.

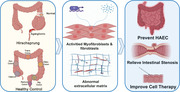

## 1 INTRODUCTION

Hirschsprung's disease (HSCR) is a relatively common congenital disability (occurrence rate is about 1/5000) characterised by the absence of ganglion cells in the distal intestine.^1^ HSCR occurs when enteric nervous system (ENS) precursor cells‐enteric neural crest cells (ENCC) fail to colonise the distal fetal intestine.^2^ The current treatment strategy of HSCR is limited to pull‐through operation.^2^ However, even after surgery, HSCR‐associated enterocolitis (HAEC) remains common (∼37%), which is the most severe complication of HSCR.^3^ The pathogenesis of abnormal ENS development is still unclear, and the HAEC aetiology is poorly understood.

Recently, the role of abnormal extracellular matrix (ECM) in the pathogenesis of HSCR has attracted more and more attention. ECM plays a crucial role in the regulation of intestinal microenvironment homeostasis.^4^ ECM dysregulation affects ENCC colonisation and survival.^5^, ^6^ Moreover, abnormal ECM is associated with intestinal fibrosis in intestinal inflammation such as enterocolitis.^7^

On the one hand, previous studies found abnormal expression alterations of various ECM proteins in HSCR segments. For example, the expression and distribution of type I,^8^, ^9^ type III,^8^, ^9^ type IV^8^, ^9^, ^10^, ^11^, ^12^ and type VI^13^ collagens are altered in aganglionic segments of HSCR. Among them, type III^14^ and IV^14^, ^15^ collagen are higher expressed in animal models of HSCR. In addition, fibronectin^14^, ^16^, ^17^ and laminin^10^, ^11^, ^18^, ^19^ were up‐regulated in aganglionic segments of HSCR.

On the other hand, though, previous studies found that collagen,^6^, ^13^, ^15^, ^20^ fibronectin^21^, ^22^ and laminin^5^, ^23^ have an impact on the development of ENCC in vitro and in vivo experiments, which is associated with the pathogenesis of HSCR. Besides, stem cell transplantation experiments found that the survival time of ENCC transplanted into aganglionic segments of animal models is short,^24^ which may be related to the ECM dysregulation in aganglionic segments.

It is worth noting that abnormal deposition of ECMs in the intestinal wall of HSCR is a crucial feature of intestinal fibrosis.^25^ The essential process of intestinal fibrosis includes the activation of myofibroblast and collagen‐producing cells in the enterocolitis^25^: The abnormal proliferation of fibroblasts and other stromal cells and their activation into myofibroblasts promote massive deposition of collagen, leading to intestine thickening, intestinal stenosis, intestinal obstruction and other symptoms through transforming growth factor‐beta (TGFB) pathway and nuclear factor‐kappa B (NF‐κB) pathway.^26^, ^27^, ^28^, ^29^, ^30^, ^31^ This pathological process suggests an intestinal fibrosis phenotype in the aganglionic segments, which may be associated with stenosis in HSCR and HAEC occurrence.

In the past, the RNA‐sequencing of the HSCR intestine were conducted at the tissue level due to technical limitation, and it was not possible to explore the cellular subsets related to the ECMs production and activation of myofibroblasts in the HSCR intestine. Single‐cell RNA‐sequencing (scRNA‐seq) can distinguish cellular subsets and analyse the cellular heterogeneity and regulation mechanism of abnormal ECMs expression in aganglionic segments.

This study systematically explores the alterations of ECMs and their mechanism in HSCR at single‐cell resolution, which provides new insight into the pathogenesis of HSCR and HAEC and new ideas for the prevention of HAEC and improvement of stem cell therapy. First, the scRNA‐seq was performed on the 8 aganglionic and 5 normal segments from 7 HSCRs and then combined with the scRNA‐seq data within 26 healthy segments from 7 healthy donors to define the cellular subsets and identify cells with an active ECM gene set. Second, the alterations of ECM‐related gene expression and the trajectory of myofibroblasts activation were analysed in stromal and glial subsets among different segments. Third, this study inferred and validated the fibrosis‐related regulators and transcription factors to further elucidate the intestinal fibrosis phenotype in aganglionic segments of HSCR.

## 2 RESULTS

### 2.1 Integrated map of HSCR and healthy control cells

**FIGURE 1:**
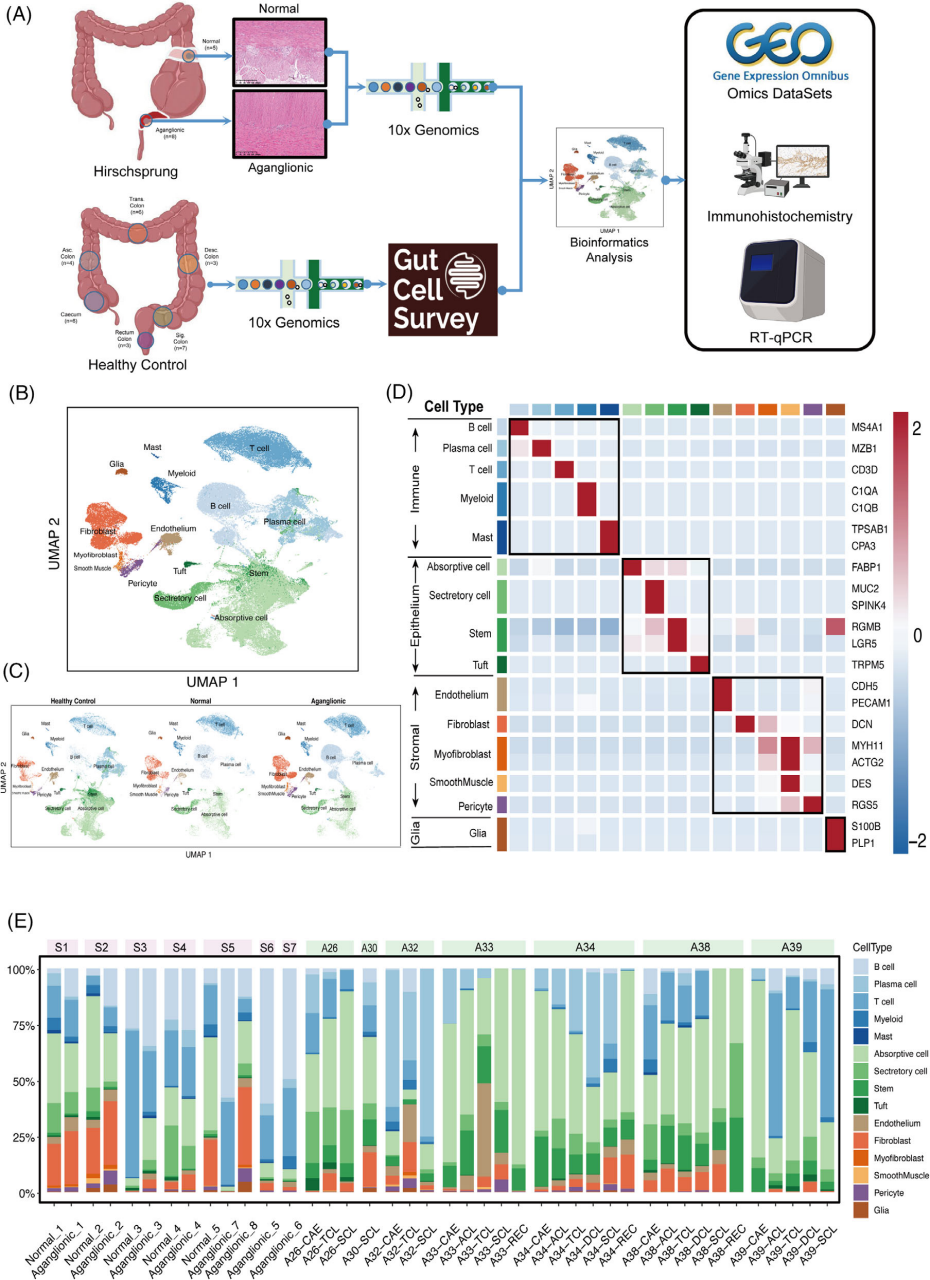
Single‐cell profiling of Hirschsprung's disease (HSCR) and healthy control intestine. (A) Schematic illustration of experimental design. Blue circles mark the biopsy location. (B) and (C) uniform manifold approximation and projection (UMAP) projection of HSCR (*n* = 7 subjects)/healthy control (*n* = 7 donors), and display separately healthy control, normal, aganglionic segments single‐cell RNA‐sequencing (scRNA‐seq) samples, respectively. Created with BioRender.com. (D) Heatmap of relative expression of cells expressing marker genes in merge datasets. The colour bars match the cell‐type group colours. (E) Changes in cell type abundance (% of cells) among different intestinal segments grouped by subjects. Subjects’ annotations coloured in pink and green are HSCR and healthy control, respectively. 10X Genomics, 10X Chromium device; RT‐qPCR, real‐time polymerase chain reaction.

To systematically investigate the alterations of ECM in HSCR, 5 normal segments and 8 aganglionic segments from 7 HSCR resections (2 short‐HSCR, 1 common‐HSCR, 1 long‐HSCR and 3 total colonic aganglionosis [TCA]) were dissociated into single‐cell suspensions and analysed using single‐cell RNA‐seq (Figure 1A, Table S1). A total of 92 958 individual cells (59 594 in aganglionic segments and 33 364 in normal segments) were used in the analysis after passing quality control and doublet exclusion criteria. In addition, 64 045 individual cells from 7 healthy donors (6 caecums, 4 ascending colons, 6 transverse colons, 3 descending colons, 7 sigmoid colons and 3 rectums) were downloaded from the Gut Cell Atlas and combined with HSCR data for further analysis (Figure 1A, Table S1).

Following dimensional reduction and visualisation with uniform manifold approximation and projection (UMAP), a two‐stage annotation strategy was used in this study. In the first stage, we used canonical genes to annotate each cell by identifying major cellular compartments, including stromal (fibroblasts, myofibroblasts, smooth muscle cells, pericytes and endothelium), glia, immune (B cells, plasma cells, T cells, myeloid and mast) and epithelium (absorptive cells, secretory cells, stem cells and tuft) cells—with HSCR and healthy segments (Figure 1B–D). In the second stage, we extracted and re‐clustered ECM‐related cellular compartments and used known morphological marker genes to annotate subclusters (Table S2).

In this study, we focused on the ECM‐related cellular compartment (especially for stromal cells) to explore the alterations of the ECM. It is worth noting that a larger proportion of fibroblast was observed in the aganglionic segments than normal segments in subjects 1, 2, 3, 4 and 5 (Figure 1E). To avoid confounding bias due to the intestinal location, cellular proportion in the distal colon and the proximal colon per healthy subject was also considered. The alterations of fibroblast proportion described above did not show a similar trend in healthy subjects (Figure 1E).

Notably, the fibroblast is the predominant producer of ECM, and an abnormal increase of fibroblasts contributes to the fibrosis in various tissues,^25^, ^32^ which infers that there is fibrosis in the HSCR segments. Hence, we next aimed to elucidate the fibrotic alterations and mechanisms further.

### 2.2 Origin and alteration of ECM in HSCR

**FIGURE 2:**
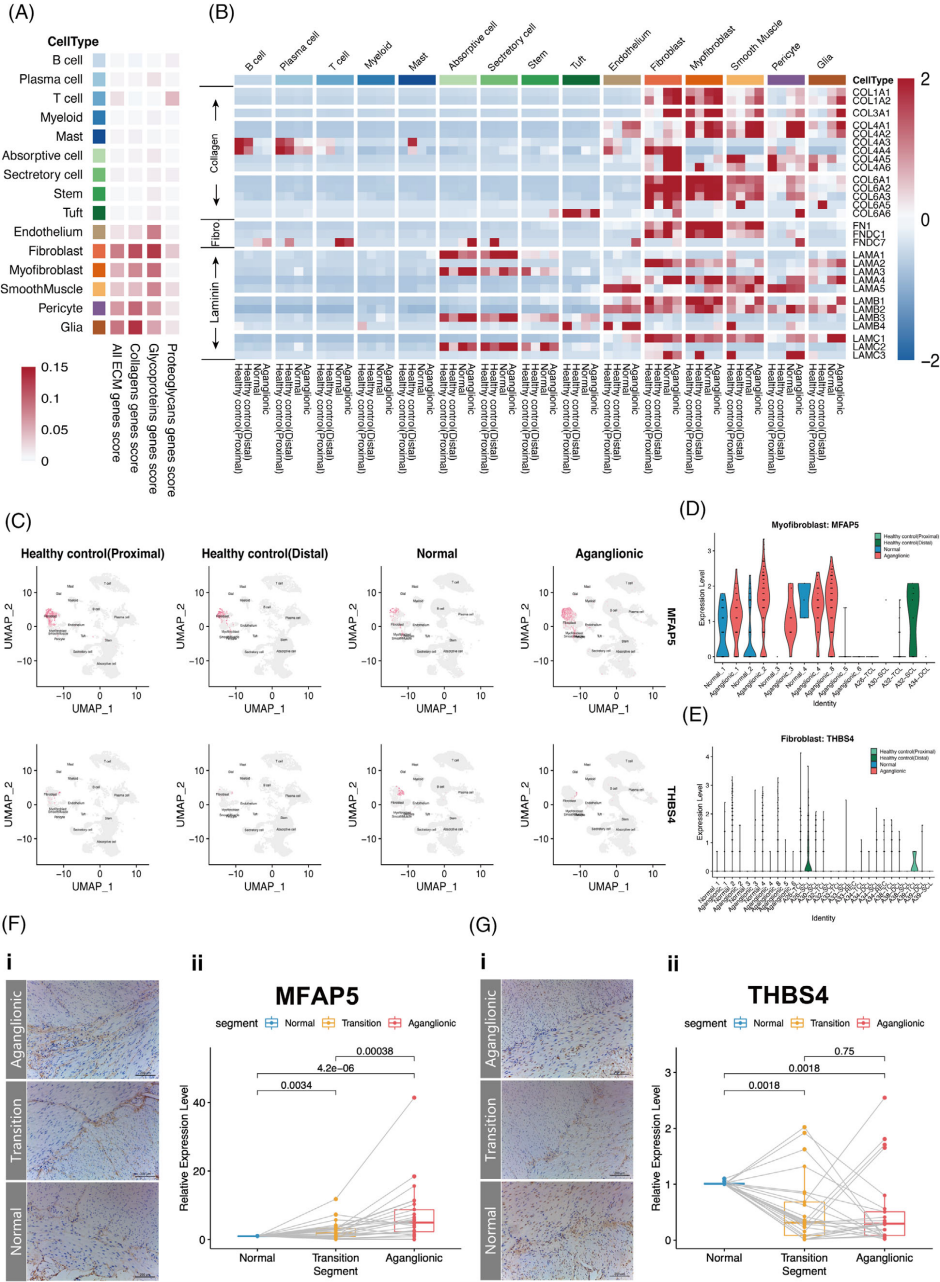
Source of extracellular matrix (ECM) in the intestine. (A) Heatmap of area under the curve (AUC) scores of collagens, glycoproteins, proteoglycans and all ECM gene set in merge datasets. The colour bars match the cell‐type group colours. (B) Heatmap of HSCR‐related ECM genes among different segments in merge dataset. The colour bars match the cell‐type group colours. (C) FeaturePlot of *MFAP5* and *THBS4* among different segments. (D) Violin plot of expression of *MFAP5* among different samples in smooth muscle, myofibroblast and fibroblast. (E) Violin plot of expression of *THBS4* among different samples in smooth muscle, myofibroblast and fibroblast. (F) Representative images of immunohistochemistry (IHC) for MFAP5 protein in the enteric ganglia and smooth muscle layers of the normal and aganglionic sections (i). Real‐time polymerase chain reaction (RT‐qPCR) validation of the *MFAP5* gene in the normal and aganglionic segments per subject, *p*‐value was calculated from Wilcoxon matched‐pairs signed‐rank test (ii). (G) Representative images of IHC for THBS4 protein in the enteric ganglia and smooth muscle layers of the normal and aganglionic sections (i). RT‐qPCR validation of the *THBS4* gene in the normal and aganglionic segments per subject, *p*‐value was calculated from Wilcoxon matched‐pairs signed‐rank test (ii). UMAP, uniform manifold approximation and projection.

To identify the cell types that contribute to the production of ECM in HSCR, we established a single‐cell ECM expression score that included collagens, glycoproteins and proteoglycans. We confirmed an increased score in fibroblasts, myofibroblasts, smooth muscle, pericytes and glial cells (Figures 2A and S1A–D).

Among myofibroblasts, smooth muscle cells, pericytes and glial cells, ECM scores demonstrated a clear shift towards higher ECM‐expressing cells in aganglionic segments (Figure S2A–C). In order to explore the abnormal changes in HSCR‐related ECM reported in previous studies,^8^, ^9^, ^10^, ^11^, ^12^, ^14^ the HSCR‐related ECM protein‐coding genes were selected from the ECM protein‐coding gene set, including type I, III, IV and VI collagen, fibronectin and laminin, and the average expression level among the cell subsets was calculated. Consistent with the ECM expression score, the relevant coding genes are mainly highly expressed in fibroblasts, myofibroblasts, smooth muscle cells, pericytes and glial cells (Figure 2B).

To further verify the abnormal expression alterations of ECM proteins (type I, III, IV and VI collagen, fibronectin, laminin) in previous studies at the single‐cell level, we further calculated the relative expression levels of major coding genes in cellular subsets of HSCR intestine for the first time. Compared with normal and healthy segments, type I, III, IV and VI collagen‐coding genes (*COL1A1*, *COL1A2*, *COL3A1*, *COL4A1*, *COL4A2*, *COL6A1*, *COL6A2*, *COL6A3*) are highly expressed in fibroblasts, myofibroblasts, smooth muscle cells, pericytes and glia subsets in aganglionic segments (Figures 2D and S1D). Fibronectin‐coding genes *FN1* and *FNDC1* are highly expressed in fibroblast, myofibroblasts and smooth muscle cells subsets of HSCR intestinal segments, compared to healthy segments. Laminin‐coding genes (*LAMA2*, *LAMA4*, *LAMA5*, *LAMB1*, *LAMB2*) are highly expressed in smooth muscle cells and glial cells subsets in HSCR intestinal segments (Figures 2B and S1F).

ECM protein‐coding genes such as collagen and laminin are abnormally highly expressed in subsets such as fibroblasts and myofibroblasts in aganglionic segments, suggesting aganglionic segments is characterised by intestinal fibrosis. In addition, there are a lot of ECM‐coding genes that are abnormally expressed in the aganglionic segment (Figure S2A–C). After considering factors such as statistical difference, biological functional associated with HSCR and antibody purchase feasibility, we selected two of the genes of HSCR phonetype‐related, *MFAP5* and *THBS4*, for further validation. *MFAP5* is abnormally highly expressed in myofibroblasts and smooth muscle cells, while *THBS4* is abnormally low in fibroblasts (Figure 2C–E). Immunohistochemistry (IHC) analysis of intestinal sections from HSCR showed that MFAP5 protein and THBS4 protein are expressed in the ECM around enteric ganglia (between the smooth muscle layers of the intestine; Figure 2F,i,G,i). It is worth noting that the alterations of *MFAP5* and *THBS4* relative expression levels were consistent with scRNA‐seq results, even in the transition segments (Figure 2F(ii),G(ii)).

*MFAP5*‐high fibroblast was previously reported to drive fibrosis in systemic sclerosis‐associated interstitial lung disease,^33^ infrapatellar fat pad,^34^ and ovarian and pancreatic cancer,^35^ which suggests that there may be intestinal fibrosis in the HSCR aganglionic segments. Previous studies supported that MFAP5^36^ and THBS4^37^ from cancer‐associated fibroblasts associated with the invasion and migration of cancer cells. The ENCC migrates extensively during embryogenesis using strategies reminiscent of those observed during cancer metastasis.^38^ The alteration of MFAP5 protein and THBS4 protein from fibroblast might be associated with the migration of ENCC—similar to the cancer cells.

It is worth noting that stromal cells and glial cells are the primary sources of ECM, and we further explored the heterogeneity of these cells in HSCR.

### 2.3 The cellular source of myofibroblasts

**FIGURE 3:**
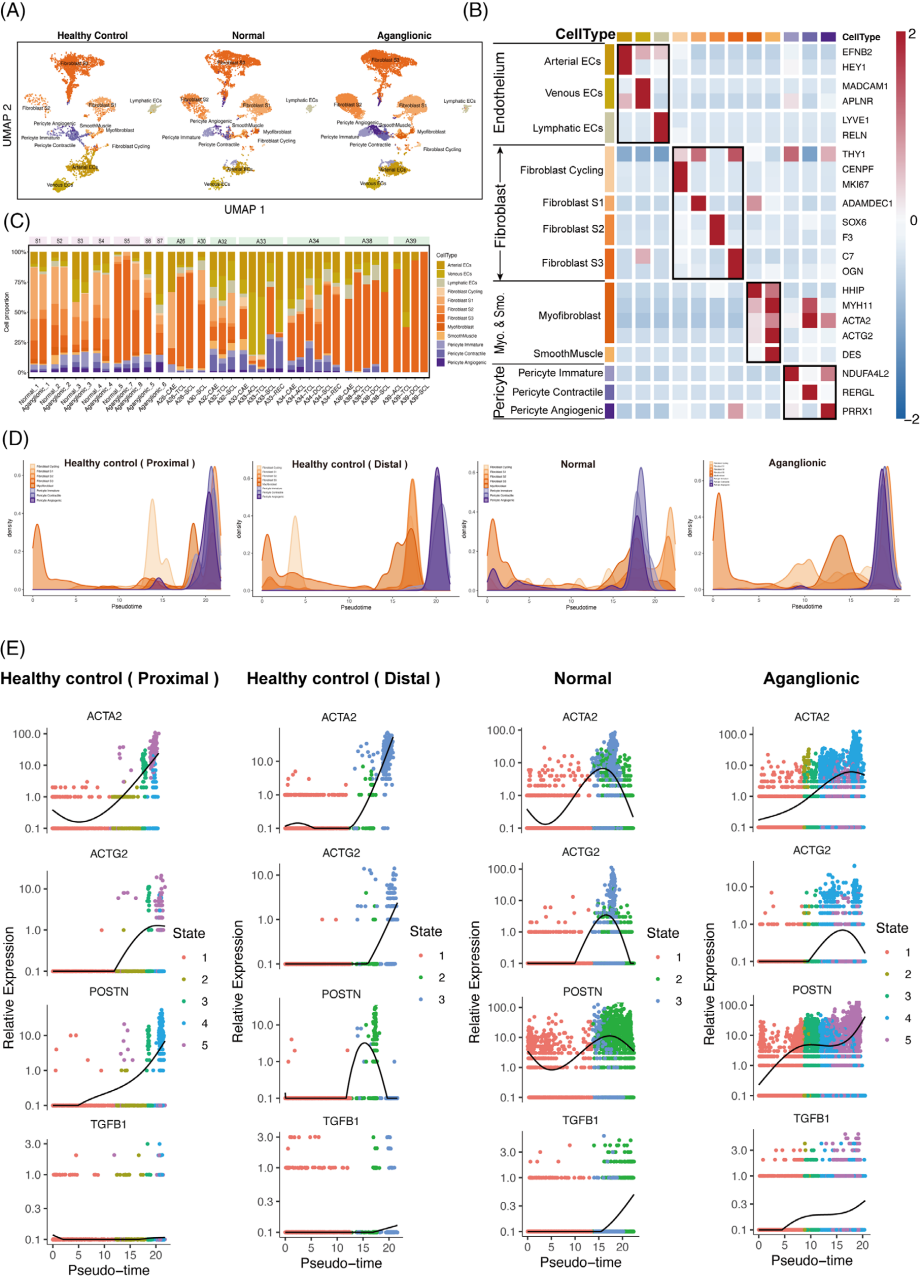
Origin of myofibroblasts. (A) uniform manifold approximation and projection (UMAP) of stromal cells grouped by healthy control, normal, aganglionic segments. (B) Heatmap of relative expression of cells expressing marker genes in stromal cells. The colour bars match the cell‐type group colours. (C) Changes in cell type abundance (% of cells) among different intestinal segments grouped by subjects. Subjects’ annotation coloured in pink and green are HSCR and healthy control, respectively. (D) Cell density map display the cell density of different cell types along the trajectory. (E) Scatter plot display the expression levels of myofibroblast marker *ACTA2* & *ACTG2*, fibrosis‐associated myofibroblast markers POSTN, myofibroblast activation regulator TGFB1 along the trajectory.

In order to explore the molecular mechanism of abnormal changes in ECM, 23 369 stromal cells, including 11 961 aganglionic segment cells, 5147 normal segment cells and 5223 healthy control cells were extracted for in‐depth annotation and further analysis (Figure 3A). According to the expression of cell markers, four types of 12 cell subsets were further annotated (Figure 3B). Compared to the healthy intestine, the proportion of proliferating fibroblasts and myofibroblasts was larger in the HSCR intestine (Figure 3C). Increased numbers of fibroblasts and myofibroblasts suggest fibrotic changes in the HSCR intestine.^25^, ^32^ The alterations of HSCR‐related ECM protein‐coding genes were similar in the ECM‐related cellular subsets (Figure S3A,B).

Previous studies have confirmed that myofibroblasts are activated fibroblasts associated with abnormal ECM protein expression and deposition.^39^, ^40^ In addition, pericytes are also sources of myofibroblasts.^41^ In order to explore the differentiation pattern of myofibroblasts among different segments, fibroblasts, myofibroblasts and pericyte subsets were extracted for pseudotime cell ordering inference (Figure S3C,D).

Earlier differentiation of myofibroblasts occurred in aganglionic segments, compared to healthy and HSCR normal segments (Figure 3D). The expression changes of the myofibroblast marker *ACTA2* and *ACTG2*,^42^ fibrosis‐related growth factor *POSTN* ^43^, ^44^ and the myofibroblast regulatory factor *TGFB1* ^25^ with pseudotime cell ordering were further observed (Figures 3E and S3E). *ACTA2* and *ACTG2* was expressed at an earlier stage in HSCR aganglionic segments, compared with healthy and HSCR normal segments, suggesting more pronounced fibroblast activation in aganglionic segments (Figures 3E and S3E). *POSTN* was over‐expressed at an earlier stage in HSCR normal and aganglionic segments, indicating a tendency for fibrosis in HSCR segments, especially in aganglionic segments (Figures 3E and S3E). The expression of the myofibroblast regulator *TGFB1* increased over time in normal and aganglionic segments, and *TGFB1* expression appeared at an earlier stage in aganglionic segments (Figures 3E and S3E). It suggested that more fibroblasts and pericytes in the aganglionic segment differentiated into myofibroblasts at an earlier stage, and these cells promote intestinal fibrosis.

### 2.4 Heterogeneity of the ECM‐related stromal cell in the aganglionic segment

To further explore the alterations in ECM‐related stromal cells (fibroblasts, myofibroblasts and pericytes) in aganglionic segments, we performed paired and unpaired differential analysis at single‐cell transcriptomes, bulk transcriptomes and proteomes. Considering the up‐regulated ECM coding gene expression in aganglionic segments, we focused on the up‐regulated differentially expressed genes (DEGs) in the HSCR segments.

The intersections of up‐regulated DEGs associated with the aganglionic segments (HSCR aganglionic segment vs. HSCR normal segment, HSCR aganglionic segment vs. healthy distal segment) at single‐cell transcriptomes were taken and used for Gene Ontology (GO) enrichment analysis. The detailed DEGs list of different cell types can be found in Table S3.

**FIGURE 4:**
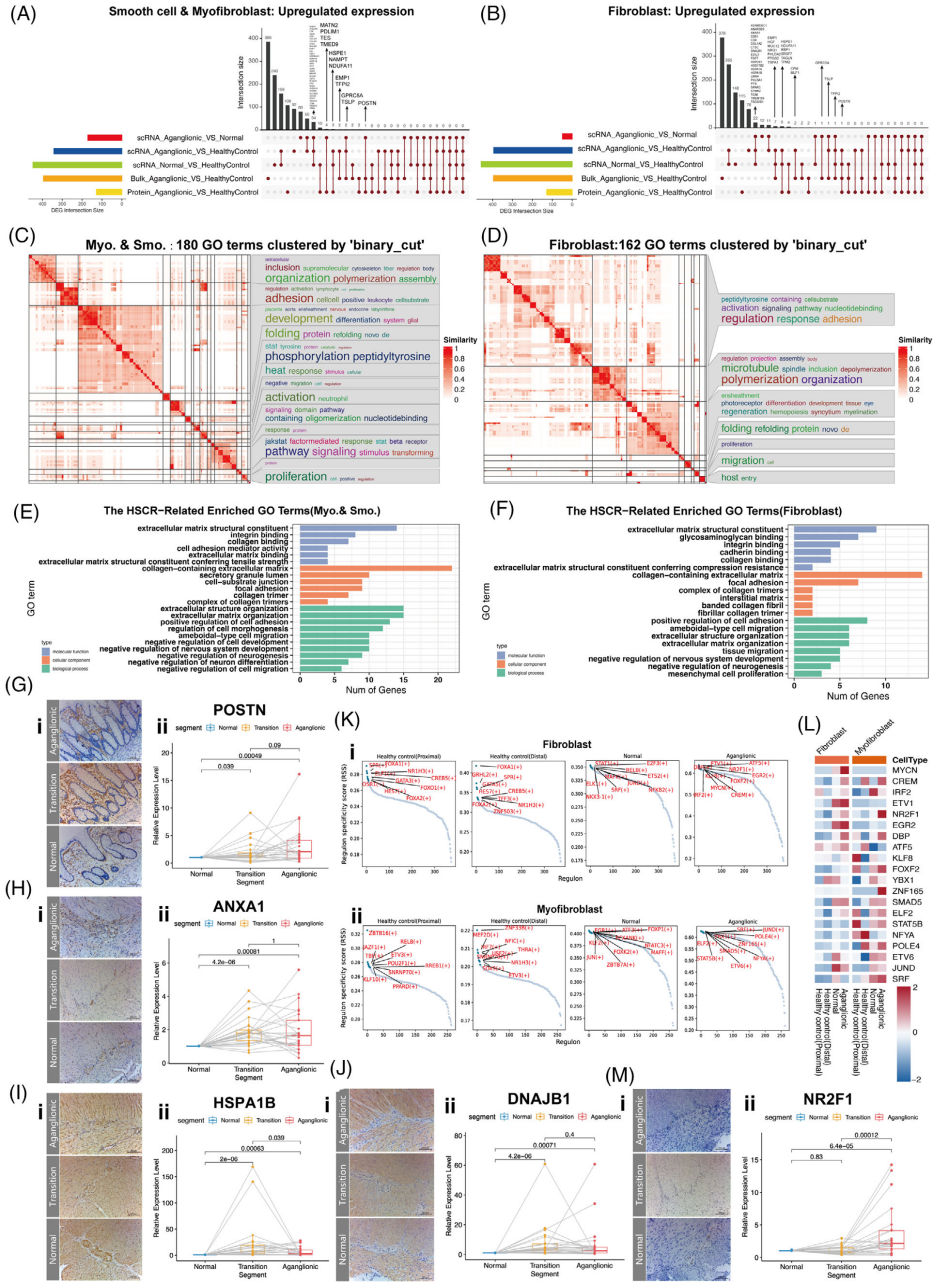
Heterogeneity of ECM‐related stromal cell. (A)Static UpSet plot of the up‐regulated differentially expressed genes (DEGs) among different set intersections in the myofibroblasts and smooth muscle cells subset. (B) Static UpSet plot of the up‐regulated DEGs among different set intersections in the fibroblasts subset. (C) Heatmap of simplying the gene ontology (GO) enrichment results in the myofibroblasts and smooth muscle cells subset by clustering the significant GO terms, on the right side of the heatmap there are the word cloud annotations which summarise the functions with keywords in every GO cluster. (D) Heatmap of simplying the GO enrichment results in the fibroblasts subset by clustering the significant GO terms; on the right side of the heatmap, there are the word cloud annotations that summarise the functions with keywords in every GO cluster. (E) Barplot of HSCR‐related significantly GO terms in the myofibroblasts and smooth muscle cells subset. (F) Barplot of HSCR‐related significantly GO terms in the fibroblasts subset. (G) Representative images of IHC for POSTN protein in the mucosa layers of the normal and aganglionic sections (i). RT‐qPCR validation of the *POSTN* gene in the normal and aganglionic segments per subject, *p*‐value was calculated from Wilcoxon matched‐pairs signed‐rank test (ii). (H) Representative images of IHC for ANXA1 protein in the enteric ganglia and smooth muscle layers of the normal and aganglionic sections (i). RT‐qPCR validation of the *ANXA1* gene in the normal and aganglionic segments per subject, *p*‐value was calculated from Wilcoxon matched‐pairs signed‐rank test (ii). (I) Representative images of IHC for HSPA1B protein in the enteric ganglia and smooth muscle layers of the normal and aganglionic sections (i). RT‐qPCR validation of the *HSPA1B* gene in the normal and aganglionic segments per subject, *p*‐value was calculated from Wilcoxon matched‐pairs signed‐rank test (ii). (J)Representative images of IHC for DNAJB1 protein in the enteric ganglia and smooth muscle layers of the normal and aganglionic sections (i). RT‐qPCR validation of the *DNAJB1* gene in the normal and aganglionic segments per subject, *p*‐value was calculated from Wilcoxon matched‐pairs signed‐rank test (ii). (K) Regulon specificity scores (RSS) panel plot of top 10 regulons with all segments in the myofibroblasts subset (i) and fibroblasts subset (ii). (L) Heatmap of top 10 regulons among different segments in myofibroblasts and fibroblasts subset. The colour bars match the cell‐type group colours. (M) Representative images of IHC for NR2F1 protein in the enteric ganglia and smooth muscle layers of the normal and aganglionic sections (i). RT‐qPCR validation of the *NR2F1* gene in the normal and aganglionic segments per subject, *p*‐value was calculated from Wilcoxon matched‐pairs signed‐rank test (ii).

There were 86 up‐regulated DEGs in the intersection of myofibroblasts and smooth muscle cells subsets (Figure 4A). Hundred and eighty GO terms were clustered and indicated that DEGs are involved in biological processes related to HSCR phenotype, such as ECM organisation, negative regulation of nervous system development and negative regulation of cell migration (Figure 4C,E). The results showed that 35 up‐regulated DEGs were in the intersection of fibroblasts (Figure 4B), and the DEGs are also involved in the similar biological processes described above (Figure 4D,F). There were 39 up‐regulated DEGs in the intersection of pericytes (Figure S4A), but DEGs are not involved in HSCR‐related biological processes (Figure S4B,C).

To further validate the DEGs at the RNA and protein levels, we analysed transcriptomic and proteomic data to verify that the same changes occurred (Figure 4A). It is worth noting that the *POSTN* in the intersection is involved in regulating tissue fibrosis and cell migration (Figure 4A,B). Fibrosis‐related secretory protein POSTN showed significantly up‐regulated in aganglionic segments among single‐cell transcriptomes, bulk transcriptomes and proteomes in fibroblast, myofibroblasts and smooth muscle cells subsets. IHC for POSTN showed a localisation pattern around crypts in the intestinal mucosa of HSCR segments, which is identical to inflammatory bowel disease (IBD^45^; Figure 4G(i)). The changing trend of *POSTN* in aganglionic segments is consistent with the scRNA‐seq results (Figure 4G(ii)). POSTN is a fibrogenic growth factor that drives fibrosis pathogenesis.^43^ Previous studies^46^ suggested that POSTN was increased and could promote collagen production in keloid (a fibroproliferative disorder). Furthermore, *POSTN* induces intestinal inflammation by activating NF‐κB signalling,^47^ possibly related to HAEC.

Some DEGs in the intersection of single‐cell transcriptomes are also involved in ECM synthesis and tissue fibrosis. For example, *ANXA1* is abundantly expressed in the aganglionic segments in fibroblast, myofibroblasts and smooth muscle cells and pericytes subsets and included in most of the significantly enriched GO terms related to the HSCR pathogenesis (Figures 4A–F and S4A–C). IHC showed that ANXA1 protein is expressed in and around enteric ganglia in the HSCR segments (Figure 4H(i)), and the relative expression alteration of *ANXA1* in aganglionic segments is consistent with the scRNA‐seq results (Figure 4H(ii)). Previous studies^48^ found that ANXA1 protein modulates fibroblast phenotype and ECM synthesis activity. Heat shock 70 protein‐coding gene *HSPA1B*, *HSPA1A* and heat shock 40 protein‐coding gene *DNAJB1* were up‐regulated in the aganglionic segments in fibroblast, myofibroblasts and smooth muscle cells and pericytes subsets (Figures 4A,B and S4A). IHC showed that *HSP70* protein was expressed in and around enteric ganglia, but HSP40 protein was expressed out of enteric ganglia (Figure 4i i,4i i). The relative expression level of *HSPA1B* and *DNAJB1* were consistent with the scRNA‐seq results (Figure 4i ii, 4i ii). Transition segments showed the same trend as the aganglionic segments (Figure G–J(ii)). Notably, HSP70 protein is up‐regulated in keloid fibroblasts and tissue^49^, ^50^ and atherosclerosis.^51^ On the one hand, up‐regulated genes in fibroblast, myofibroblasts and smooth muscle cells subsets in the aganglionic segments were associated with functions such as ECM formation and construction, collagen binding and cell adhesion, suggesting that DEGs are involved in abnormal ECM expression. On the other hand, genes such as *POSTN*, *ANXA1* and *HSPA1B* were highly expressed in the aganglionic segments, and previous studies confirmed they are involved in fibroblast activation and tissue fibrosis. Both pseudotime cell ordering inference and differential analysis indicated that fibroblasts and myofibroblasts were involved in abnormal ECM expression and tissue fibrosis.

In order to deeply understand its molecular mechanism, the transcriptional regulatory network analysis was used to infer the transcription factors involved in the regulation of ECM formation and fibrosis in related cell subsets. We calculated regulon specificity scores (RSS) across HSCR and healthy segments and selected the top 10 regulons from aganglionic segments (Figures 4K(i,ii) and S4H). The regulons expression was up‐regulated in HSCR segments(Figures 4L and S4D,E,I,J). Several regulons expression levels were significantly correlated with the expression levels of ECM‐encoding genes in related cell subsets, such as *NR2F1* in fibroblasts (Figure S4F,G,K). IHC showed that NR2F1 protein was expressed in the nucleus within the enteric ganglia and smooth muscle layers in the HSCR segments (Figure 4M(i)), and the up‐regulated *NR2F1* expression in aganglionic segments was consistent with scRNA‐seq results (Figure 4M(ii)). Transition segments showed the same trend as the aganglionic segments (Figure 4M(ii)). It is worth noting that transcription factor NR2F1 protein is involved in regulating ECM expression and tissue fibrosis in silicosis.^52^ In addition, NR2F1 protein is a known tumour dormancy marker in cancer‐associated fibroblasts.^53^

The abnormal expression of fibrosis‐related regulatory factors and transcription factors in ECM‐related stromal cells indicate that there is a fibrotic phenotype in the HSCR segments, especially in the aganglionic segments. These factors may be involved in the regulation of intestinal fibrosis.

### 2.5 Heterogeneity of the glial cell in the aganglionic segment

**FIGURE 5:**
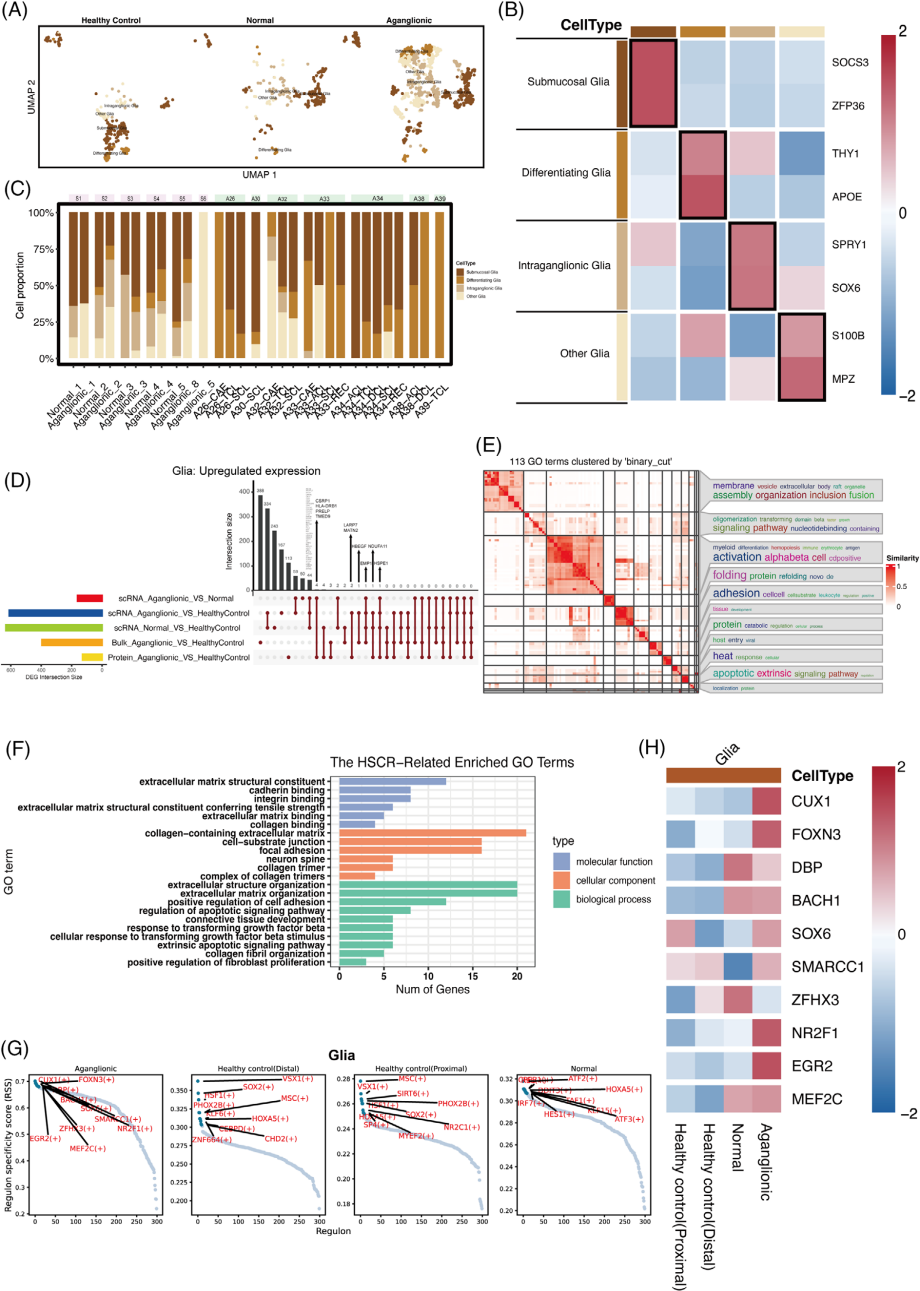
Heterogeneity of glial cell. (A) UMAP of glial cells grouped by healthy control, normal, aganglionic segments. (B) Heatmap of relative expression of cells expressing marker genes in glial cells. The colour bars match the cell‐type group colours. (C) Changes in cell‐type abundance (% of cells) among different intestinal segments grouped by subjects. Subjects’ annotation coloured in pink and green are HSCR and healthy control, respectively. (D) Static UpSet plot of the up‐regulated DEGs among different set intersections in the glial subset. (E) Heatmap of simplying the GO enrichment results in the glials subset by clustering the significant GO terms; on the right side of the heatmap, there are the word cloud annotations that summarise the functions with keywords in every GO cluster. (F) Barplot of HSCR‐related significantly GO terms in the glials subset. (G) RSS panel plot of top 10 regulons with all segments in the glials subset. (H) Heatmap of top 10 regulons among different segments in glials subset. The colour bars match the cell‐type group colours.

The above analysis found that glial cells are also the primary source of ECM, and glial cells subsets were extracted for in‐depth annotation for subsequent analysis—a total of 1265 glial cells, including 810 aganglionic segment cells, 205 normal segment cells and 240 healthy segment cells (Figure 5A). Four glial cells subsets were further annotated according to the expression of cell markers (Figure 5B). A higher proportion of intraganglionic glial cells in HSCR segments were compared to healthy segments (Figure 5c).

Considering the up‐regulated ECM coding gene expression in aganglionic segments, we focused on the up‐regulated DEGs in the HSCR segments. The intersections of up‐regulated DEGs associated with the aganglionic segments (HSCR aganglionic segment vs. HSCR normal segment, HSCR aganglionic segment vs. healthy distal segment) at single‐cell transcriptomes were taken and used for GO enrichment analysis.

The results showed that 117 DEGs were up‐regulated at the glial cells intersection (Figure 5D). Notably, up‐regulated genes in glial cells subsets were associated with ECM organisation, suggesting that DEGs are involved in abnormal ECM expression (Figure 5E,F). In addition, some up‐regulated DEGs (e.g., *HSPA1B* and *HSPA1A*) were also associated with the regulation of the apoptotic signalling pathway, which may involve in the process of ganglion cell apoptosis in the aganglionic segments.

Interestingly, some DEGs (e.g., *ANXA1*, *HSPA1B* and *HSPA1A*) are also present in the intersection of single‐cell transcriptomes in the glia subset, which may be involved in intestinal fibrosis (Figure 5D–F). IHC showed that ANXA1 protein and HSP70 protein were expressed in the enteric ganglia in the HSCR segments (Figure 4H(i),I(i)), and the expression alterations in aganglionic segments were consistent with scRNA‐seq results (Figure 4H(ii),I(ii)). Differential analysis and functional enrichment analysis suggested that glial cells subsets were involved in abnormal expression of ECM and tissue fibrosis (Figure 5E,F).

The transcriptional regulatory network analysis was conducted to explore the transcription factors involved in regulating ECM formation and fibrosis in glial subsets. It is worth noting that transcription factor *NR2F1* in glial cells subsets was also involved in regulating ECM expression and tissue fibrosis in aganglionic segments (Figure 5G). The regulon *NR2F1* expression was up‐regulated in aganglionic segments (Figures 5H and S5A,B) and significantly correlated with the expression levels of ECM‐encoding genes in glial cells (Figure S5C). IHC showed that NR2F1 protein was expressed in the nucleus within the enteric ganglia in the HSCR segments (Figure 4M(i)), and the expression alteration was consistent with scRNA‐seq results (Figure 4M(ii)). Interestingly, previous studies^54^ found that up‐regulated *NR2F1* expression in a mouse model results in a megacolon phenotype.

These fibrosis‐related regulatory factors and transcription factors in the glial subset may be involved in regulating intestine fibrosis in the aganglionic segments.

### 2.6 Expression changes linked to known HSCR‐associated genes

**FIGURE 6:**
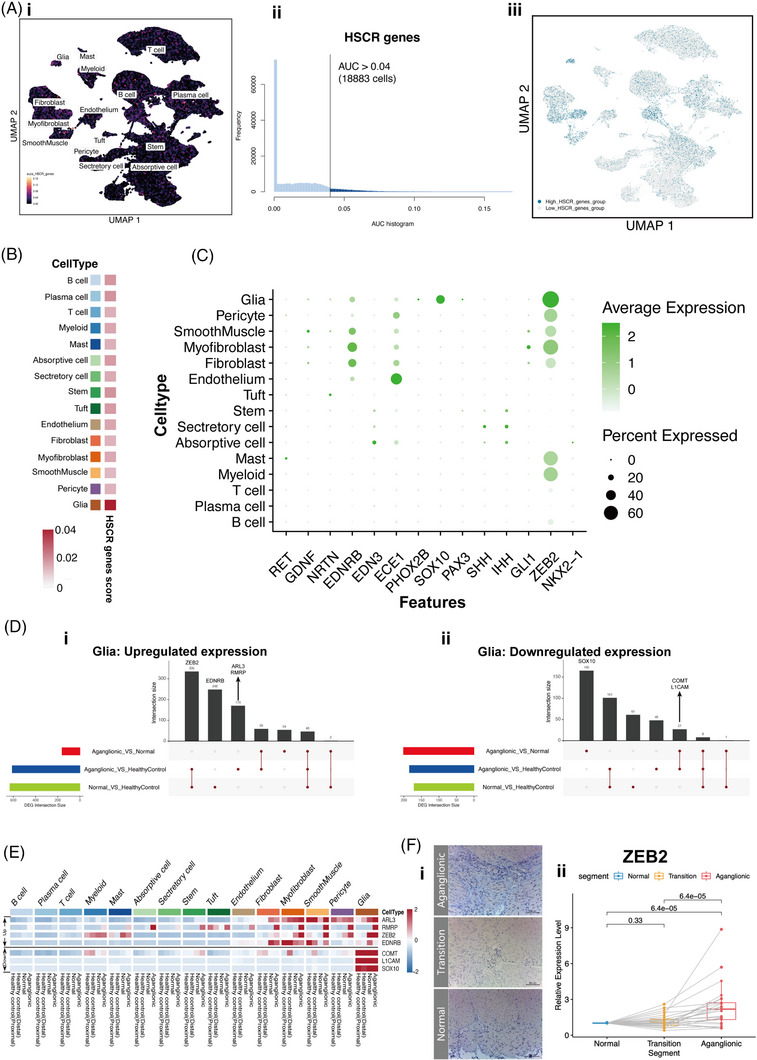
The expression pattern of known HSCR‐associated genes. (A) UMAP is coloured based on the AUC scores of known HSCR‐associated gene set (i). The AUC histogram of HSCR‐associated gene set, the grey line is the threshold suggested for the HSCR‐associated gene‐set (ii).To highlight the cluster of cells that are more likely of the cell type according to the signatures, we split the cells into the cells that pass the assignment threshold (coloured in shades of blue) and the cells that do not (coloured in light grey) in the UMAP (iii). (B) Heatmap of AUC scores of known HSCR‐associated gene set grouped by segments. The colour bars match the cell‐type group colours. (C) Dot plots of relative expression and percentage of cells expressing 14 recognised key pathogenic genes in datasets. (D) Static UpSet plot of the up‐regulated and down‐regulated DEGs among different set intersections in the glial subset. (E) Heatmap of relative expression of cells expressing intersection of known HSCR‐associated genes in merge datasets. (F) Representative images of IHC for ZEB2 protein in the enteric ganglia and smooth muscle layers of the normal and aganglionic sections (i). RT‐qPCR validation of the *ZEB2* gene in the normal and aganglionic segments per subject*, p*‐value was calculated from Wilcoxon matched‐pairs signed‐rank test (ii).

Due to the limitation of the scRNA‐seq platform, it is impossible to analyse the enteric neurons directly; we further observed the expression changes of HSCR‐related genes in the other cellular subsets. Hundred and eleven known HSCR‐associated genes were collected from The Human Phenotype Ontology website (https://hpo.jax.org/app/). We performed the HSCR‐associated gene expression score for all subsets (Figure 6A) and confirmed an increased score in glial cells (Figure 6B). Some major HSCR‐associated genes (*ZEB2*, *SOX10*, *PHOX2B*, *PAX3* and *EDNRB*) are mainly expressed in glial cells (Figure 6C).

We further explored the DEGs (aganglionic vs. healthy control; normal vs. healthy control). The intersections across the DEG datasets were calculated and visualised (Figure 6D,E). *ZEB2* was highly expressed in glial cells and stromal subsets in HSCR aganglionic segments (Figure 6E). IHC showed that *ZEB2* was expressed in the nucleus within the enteric ganglia and smooth muscle layers in the HSCR segments (Figure 6F(i)), and the altered *ZEB2* expression in aganglionic segments was consistent with scRNA‐seq results, even in the transition segments (Figure 6F(ii)).

*ZEB2* encodes a transcription factor that is crucial to direct the development, migration, and differentiation of ENCC.^55^ In addition, *ZEB2* is a crucial modulator of the epithelial‐to‐mesenchymal‐transition process^56^ and regulates fibrotic pathways such as the TGF β signalling pathway.^57^

## 3 DISCUSSION

There is a knowledge gap regarding the heterogeneity of ECM‐related cellular subsets and the alteration and regulation of the ECM expression in HSCR aganglionic segments at the single‐cell level. To explore the alterations of ECM and its regulatory mechanism in the HSCR intestine, we performed scRNA‐seq on 13 intestinal tissues of seven HSCRs. We then compared them with 29 colon tissues of seven healthy donors from the Gut Cell Survey database. The scRNA‐seq data were combined for bioinformatics analysis to explore the abnormal ECM expression and the mechanism of intestinal fibrosis in aganglionic segments.

Aberrant expression of ECM proteins in aganglionic segments has been demonstrated associated with HSCR phenotypes such as ENCC development and migration.^5^, ^6^, ^13^, ^15^, ^20^, ^21^, ^22^, ^23^ In addition, previous studies have found abnormal expression of ECM proteins^7^, ^58^ and fibrotic regulators in intestinal fibrosis, and myofibroblast activation is also an essential manifestation of intestinal fibrosis.^49^, ^50^, ^59^

Here, we provide evidence in HSCR that there is a fibrotic phenotype in aganglionic segments by comprehensively exploring the heterogeneity of the ECM‐related stromal cells and glial cells in the HSCR segment. We found that the ECM proteins mainly originated from fibroblasts, myofibroblasts, smooth muscle cells, pericytes and glial cells, and the ECM alterations were consistent with previous studies. The fibrosis‐related ECM coding gene MFAP5 was up‐regulated in the myofibroblasts and smooth muscle cells. The number of fibroblasts and myofibroblasts in the aganglionic segments increased. More myofibroblasts are activated at an earlier stage in aganglionic segments, and the earlier and higher expression of the myofibroblast marker *ACTA2* and *ACTG2*, fibrosis‐related marker *POSTN* and regulatory factor *TGFB1* suggested marked fibroblast activation in HSCR segments, especially in aganglionic segments. POSTN protein is a fibrogenic growth factor that drives fibrosis pathogenesis.^43^, ^44^ TGFB1 is a multifunctional profibrogenic cytokine involved in fibrosis in almost any organ and tissue, including the gastrointestinal tract.^25^ Previous studies^32^ found a fibrotic phenotype in the intestine of IBD that myofibroblasts were activated; it will promote the abnormal expression and deposition of ECM proteins in the intestine, which cause intestinal wall thickening and intestinal stenosis.^60^ This intestinal fibrotic phenotype is consistent with the alterations in HSCR aganglionic segments.

The fibrotic regulators *POSTN*, *ANXA1* and *HSP70* are highly expressed in the ECM‐related cellular subsets in the aganglionic segments and transition segments. Previous studies^46^ suggested that POSTN protein was increased and could promote collagen production in keloids. Furthermore, POSTN protein induces intestinal inflammation by activating NF‐κB signalling,^61^ possibly related to HAEC. Previous studies^48^, ^62^ have demonstrated that ANXA1 protein biosynthesis and release can be induced by the TGFB protein and exerts inhibitory effects on *ACTG2* and *COL1A1* gene expression, suggesting that *ANXA1* may be compensatively increased in intestinal fibrosis and play a role in inhibiting fibrosis in HSCR. Moreover, *ANXA1* is a glucocorticoid‐modulated anti‐inflammatory protein that plays a key role in IBD control and is a potential biomarker of IBD progression,^63^ possibly contributing to HAEC.

Previous studies have demonstrated that the *HSP70* gene may be closely associated with tissue fibrosis: High expression levels of *HSP70* have been proved that contribute to the fibrosis in fibrosis‐related diseases, including patients with keloid fibroblasts and tissue^49^, ^50^ and patients with atherosclerosis.^51^ However, up‐regulated *HSP70* also plays a role in inhibiting fibrosis.^64^ Furthermore, *HSP70* and *HSP40* are up‐regulated with IBD^65^, ^66^; their expression is significantly increased during the active stage of the disease.

The transcription factor regulatory network revealed that *NR2F1* in the fibroblasts and glial subsets was up‐regulated in the aganglionic segment and may be involved in regulating ECM expression and tissue fibrosis. Transcription factor NR2F1 protein was involved in regulating ECM expression and tissue fibrosis in silicosis—a form of pulmonary fibrosis.^52^ Besides, NR2F1 protein is a known tumour dormancy marker that promotes quiescence of various cancer cells, predominantly expressed in cancer‐associated fibroblasts,^53^ suggesting that it may intervene in the ENCC migration.

It is worth noting that the Waardenburg syndrome type 4 mouse model combines abnormal development of the ENS (causing aganglionic megacolon), which showed that ENCC‐derived ENS progenitors failed to reach the distal colon due to up‐regulated *NR2F1* inducing premature differentiation in enteric glial cells.^54^ Hence, NR2F1 protein may be an important pathogenic factor in ENCC dysplasia and fibrosis in HSCR.

All of the alterations suggest the presence of intestinal fibrosis phenotype in HSCR segments, especially in the transitional segments and aganglionic segments. This pathological change may be related to the potential regulatory mechanism. Abnormal development of enteric ENCC during the embryonic period may lead to differentiation into myofibroblasts in the HSCR intestine, which promote intestinal fibrosis. Previous studies have demonstrated that ENCC can differentiate into myofibroblasts in vitro^67^ and in vivo^68^ experiments. Second, the abnormal spasm of the aganglionic segment increases the mechanical pressure on the intestinal wall due to the loss of ganglion cells, which may promote fibrosis. Mechanical stress is also a contributing factor to intestinal fibrosis.^69^

Our results have several potential therapeutic implications. Treatment of intestinal fibrosis in the HSCR intestine will have the potential to relieve intestinal stenosis, prevent HAEC and prolong the survival of ENCC transplantation in aganglionic segments. First, after validating the fibrotic phenotype in the aganglionic segments, the inhibit the accumulation of myofibroblasts strategy can be referred to treat intestinal fibrosis in HSCR.^25^, ^70^ For example, the rho‐associated kinases inhibitor AMA0825 was promised anti‐fibrotic therapy by blocking myofibroblasts activation.^71^ Second, manipulating fibrosis regulator *POSTN*, *HSP70* and transcription factor *NR2F1* expression in the HSCR segment may be a potential therapeutic target in treating intestinal fibrosis.^25^, ^72^ Third, the treatment strategy combined with drug therapy and stromal cell intervention in the HSCR intestine is able to regulate the intestinal microenvironment while promoting survival and normal differentiation of ENCC.^25^, ^72^

In addition to these notable findings, several limitations warrant discussion. First, it is impossible to analyse the enteric neurons due to the limitation of the scRNA‐seq platform. 10X Genomics platform has become the most successful and common scRNA‐seq platform under its detection of cell number and reliability. Many essential and credible data have been published in many previous studies. For the data of this study to be widely used and shared, we chose the 10X Genomics platform for this study. It is worth noting that this study focused on exploring the regulatory mechanisms of abnormal ECM alterations in the stromal cellular subsets, which can be detected at single‐cell resolution. However, most pediatric enteric neurons are too large to be detected by the 10X Genomics platform. Moreover, the dissociation procedure also affects the enteric neurons, which are more sensitive. Enteric neurons only account for a tiny fraction of the total amount of intestinal cells. Future studies should consider adding more cells in scRNA‐seq to increase the opportunity to detect enteric neurons. Second, healthy intestinal tissues from different locations in healthy children cannot be obtained due to ethical requirements; therefore, the scope of the conclusion is limited to HSCR aganglionic segments. To further extrapolate our findings, we further combine the scRNA‐seq data and the transcriptomic and proteomic data of healthy controls in public databases to support our conclusion. Third, our study was also limited by the inability to complete immunohistological verifications of all fibrotic regulators at this time due to the absence of reliable antibodies for immunohistochemistry of the relevant markers.

## 4 CONCLUSION

Together, our results provide substantial insight into the heterogeneity of ECM‐related cells in HSCR intestines, building upon cell atlas efforts in the aganglionic, normal and healthy intestines and providing new insights into intestinal fibrosis remodelling at an unprecedented high‐resolution level. The altered expression of regulators and transcription factors now available for the ECM‐related cells add considerably to our knowledge of this crucial effector cell in intestinal fibrosis and provides new insights into their functional importance.

## 5 METHODS

### 5.1 HSCR samples

The HSCR patients were ethnic Chinese subjects collected from the Children's Hospital of Fudan University. The study protocol was approved by the Children's Hospital of Fudan University Institutional Review Board. This study was conducted according to government policies and the Helsinki Declaration. The patients enrolled in this study signed informed consent.

A total of 32 sporadic HSCR patients (24 males and six females) were recruited, and at least two pathologists examined their biopsy specimens and surgical materials. To make the diagnosis of HSCR, histological examination is characterised by the absence of ganglion cells in the myenteric and submucosal plexuses of the intestine. All forms of HSCR (short, common, long, and TCA) patients were represented in the samples according to the Chinese guideline.^73^, ^74^ We also supported the histological classification of each subject according to the UpToDate guideline (Table S1). The subjects were required not to have any other congenital malformations.

Their surgical materials were collected using normal (defined as the proximal dilated segment with ganglion cells), transition (defined as the segment between the distal dilated segment and aganglionic segment) and aganglionic (defined as the segment without ganglion cells) tissues. It is worth noting that the essential characteristic of own control is to avoid confounding factors caused by individual and regional differences. Own control combined with healthy control from public databases can control confounding factors and obtain the most reliable conclusions that meet the ethical requirements. Seven HSCR patients (five normal segments and eight aganglionic segments) were included in the scRNA‐seq, and 25 HSCR patients (25 normal segments, 25 transition segments and 25 aganglionic segments) were included in the real‐time polymerase chain reaction (RT‐qPCR). Histological classification (both Chinese guideline and UpToDate guideline), sample region, sample position, age and sex of subjects were evaluated. The detailed information on each subject in this study is shown in Table S1.

### 5.2 scRNA‐seq data of healthy controls

To control the confounding bias caused by the intestinal location, scRNA‐seq data from the Gut Cell Survey were selected for analysis after careful consideration.

First, we systematically reviewed all of the literature related to the intestine and scRNA‐seq. We found no studies involving the colon of healthy children (as of the end of this study) using scRNA‐seq. Second, sample sources must meet the principles of research ethics; fresh colon tissue from different locations in healthy children is not available. Third, in previous studies, adult scRNA‐seq data are generally used as a control group for which it is difficult to obtain healthy controls, including children with diseases,^75^, ^76^, ^77^, ^78^, ^79^ and this study focuses on the exploration of expression characteristics at the single‐cell level but does not consider changes in cell proportions with age. Finally, this study uses Harmony and SCTransform R packages to correct for differences between samples, which controls the influence of age to a certain extent.

Therefore, the healthy intestinal data from the Human Cell Atlas in the Gut Cell Atlas were finally selected.^80^ The scRNA‐seq data of 29 colon tissue samples from seven healthy people from different locations from the Gut Cell Survey website (Gut Cell Survey, https://www.gutcellatlas.org/) were download as healthy controls. See Table S2 for information.

All of the healthy scRNA‐seq data were combined for cell annotation. As for downstream analysis, the scRNA‐seq data of the sigmoid colon and rectum were extracted and defined as distal healthy control, and the scRNA‐seq data of the transverse colon and descending colon were extracted and defined as proximal healthy control according to the locations of aganglionic and normal segments in HSCR.

### 5.3 Gene sets of human matrisome

To systematically investigate the source and alteration of ECM in HSCR segments at the single‐cell level, the gene sets of human matrisome were downloaded for downstream analysis. The Comprehensive Cancer Institute of Massachusetts Institute of Technology and Harvard has compiled the genes encoding ECM proteins (http://matrisomeproject.mit.edu/).^81^ These genes can be divided into glycoproteins, collagens and proteoglycans.

### 5.4 Gene sets of HSCR‐associated genes

To explore the cellular subset associated with the HSCR in HSCR segments at the single‐cell level, we selected the genes from the Human Phenotype Ontology website (https://hpo.jax.org/app/, aganglionic megacolon HP:0002251) for downstream analysis.^82^

### 5.5 Validation by bulk transcriptomics

RNA‐seq profiles of HSCR aganglionic segments and their healthy controls were retrieved from the GSE96854 dataset in the GEO database. The above 30 colon samples for transcriptomics analysis were randomly pooled, generating three pooled healthy and three pooled aganglionic samples.^83^ The ‘limma’ package was used to validate the DEGs in the aganglionic versus healthy groups.

### 5.6 Validation by proteomics

Significantly differential proteins with changes in the expression level of HSCR aganglionic segments and their healthy controls were directly collected from the previous study. The above 30 colon samples for proteomics analysis were randomly pooled, generating three pooled healthy and three pooled aganglionic samples. The detailed analysis process can be found in the original study.^83^

### 5.7 Immunohistochemistry

The HSCR samples were excised from normal and aganglionic segment and then fixed in polyoxymethylene (4%) for at least 48 h. Fixed samples were washed and dehydrated by gradient ethanol, embedded in paraffin and sliced. To analyse the expression location of target DEGs in HSCR samples, IHC for MFAP5 (Abcam, ab203828, Rabbit monoclonal immunoglobulin G (IgG), 1:5000 dilution), THBS4 (Abcam, ab263898, Rabbit monoclonal IgG, 1:600 dilution), POSTN (Abcam, ab215199, Rabbit monoclonal IgG, 1:1000 dilution), ANXA1 (Abcam, ab214486, Rabbit monocle IgG, 1:6000 dilution), HSP70 (Abcam, ab181606, Rabbit monoclonal IgG, 1:1000 dilution), HSP40 (Cell Signaling Technology, 4871T, Rabbit monoclonal IgG, 1:50 dilution), NR2F1 (Abcam, ab181137, Rabbit monoclonal IgG, 1:100 dilution) and ZEB2 (Santa Cruz Biotechnology, sc‐271984, mouse monoclonal IgG, 1:50 dilution) was performed on 4‐μm‐thick paraffin‐embedded formalin‐fixed tissue. The GTVisionTM III Detection System/Mo&Rb kit (GENE, GK500710) was used for IHC detection according to the manufacturer's protocol. Slides were imaged using a Leica DM750 ideal microscope with the Leica ICC50 HD camera.

### 5.8 RT‐qPCR

Target DEGs were further validated by RT‐qPCR detection of intestinal samples. According to the manufacturer's manual, RNAprep Pure Tissue Kit (TIANGEN, DP431) was used to extract total RNA from HSCR intestinal samples. Total RNA was reverse transcribed into cDNA by FastKing gDNA Dispelling RT SuperMix (TIANGEN, KR118). RT‐qPCR was performed on an Applied Biosystems QuantStudio 3 Real‐Time PCR Instrument (Applied Biosystems) using an Ultra‐Rapid HotStart PCR Master Mix kit (Yeasen Biotechnology, 10157ES08). The β‐actin served as an internal control for qPCR normalisation, and the relative gene levels were calculated by the 2^−ΔΔCT^ method. The primer sequences of these genes are listed in Table S3.

### 5.9 HSCR tissue dissociation

The HSCR intestine mesentery was cut to lengthen out the normal (defined as the proximal dilated segment) and aganglionic segment tissues. Briefly, HSCR tissue samples were immediately washed twice with cold Dulbecco's Phosphate‐Buffered Saline (D‐PBS) (Gibco, 14190094) and diced with a sterile scissor. Then, the diced samples were digested in dissociation reagents containing 0.05% Trypsin, ethylene diamine tetraacetic acid, 1 mg/ml Collagenase IV, 1 mg/ml Collagenase II, 50 ug/ml deoxyribonuclease I on a shaking platform (250 rpm) at 37°C for up to 60 min according to the previous study.^84^ The single cells were filtered through a 70‐μm sieve and spun down at 800 rpm at 4°C for 5 min. Red cell lysis solution was used to remove red blood cells according to the manufacturer's guidelines. The remained cells were then re‐suspended in D‐PBS with 1% bovine serum albumin and assessed for viability and size using a Countess instrument.

### 5.10 Droplet‐based scRNA‐seq

Following digestion, single‐cell suspension for the HSCR tissue sample was processed using a 10X Chromium device (10X Genomics) as detailed by the manufacturer.^85^ Amplified cDNA and final scRNA‐seq libraries were carried out using Chromium Single Cell V3 Reagent Kit according to the manufacturer's protocol. The libraries were then pooled and sequenced on the NovaSeq 6000 (Illumina) with a depth of 400 million reads per sample.

### 5.11 Processing FASTQ files and quality control

All raw sequencing data from HSCR samples were converted to FASTQ format with Illumina bcl2fastq. FASTQ files were processed and aligned to the GRCh38‐3.0.0 human reference transcriptome with the Cellranger V3.1.0 pipeline (10x Genomics) using default parameters. Initial cell demultiplexing and gene quantification were also performed with the default 10x Cell Ranger pipeline.^86^ The resulting gene expression matrices were then processed and analysed using Seurat package V 4.0.2.^87^

Each HSCR tissue sample was filtered to remove cells with genes expressed in fewer than three cells in a sample were excluded, as well as cells that expressed fewer than 500 genes. We first removed cells with mitochondrial genes and haemoglobin genes to filter out low‐quality and potential red blood cells. A total of 13 HSCR tissue sample count matrices were merged. To account for differences in sequencing depth across samples, the SCTransform R package (Version 0.3.2)^88^ was used to normalise expression values for total unique molecular identifier counts per cell. PLCG2 gene, mitochondrial genes and ribosomal genes were removed from the highly variable genes as they contributed to the highest variability in the merged dataset according to the previous study.^89^

### 5.12 Doublet removal

In the first stage, each HSCR tissue data were filtered to remove cells with outlier cells by the distribution of gene content (with the threshold of 3000–7500 total genes) to remove potential doublets. In the second stage, Scrublet Python code (Version 0.2.3) was used to predict doublets in each HSCR tissue data according to the doublet detection pipeline,^90^ then the predicted doublets were excluded. In the final stage, the merged dataset was further annotated doublets by identifying clusters with gene expression of other clusters according to the previous study,^89^, ^91^ then the annotated doublets were excluded.

### 5.13 Dimensionality reduction, batch correction and clustering

Highly variable genes were used for the principal component analysis with Seurat function RunPCA. To remove variation of batch effect among different scRNA‐seq runs, the Harmony R package (Version 0.1.0) was used to integrate all the HSCR and healthy samples in the merged dataset with default parameters.^92^ Then, clustering was performed and visualised using uniform manifold approximation and projection (UMAP) algorithm by harmony dimensionality reduction with Seurat function RunUMAP. Stromal, immune and epithelium compartments were extracted for further analysis. For each compartment, the dimensionality reduction, batch correction and clustering analyses were conducted as described above.

### 5.14 Cell types annotation

To identify clusters in HSCR sample datasets, a two‐stage annotation strategy was used in the dataset according to the previous studies.^80^, ^91^ In the first stage, the canonical genes were used to divide the cell atlas into four cellular compartments (stromal, glial, immune and epithelium). The stromal compartment included differential expression of fibroblasts (*THY1, COL1A2, DCN*), myofibroblasts (*MYH11, ACTG2, ACTA2, DES* (−)), smooth muscle cells (*DES, MYH11, ACTG2, ACTA2*), pericytes (*RGS5*) and endothelium (*CDH5, PECAM1*). The glial compartment was annotated according to the expression of *S100B* and *PLP2*. The epithelium compartment was annotated according to the expression of *EPCAM* and *FABP1*. The immune compartment was annotated according to the expression of *PTPRC*. In the second stage, sets of known morphological marker genes were used for each compartment to annotate subclusters in deeper classification. A brief description of cell‐type annotation for each lineage is provided in Table S2, and the DEGs among different cell types are provided in Table S4. Marker gene expression was visualised using a heatmap where the colour indicates relative expression.

### 5.15 Gene set score and expression analysis

In order to explore the expression of ECM protein‐encoding genes in different intestinal cells, a gene set score was used to determine the primary source of abnormal ECM.

Identify cells with active gene sets in the merged data by the AUCell R package (Version 1.12.0).^93^ We used the ‘area under the curve’ to calculate whether a critical subset of the ECM gene sets is enriched within the expressed genes for each cell type. FeaturePlot was drawn through the ggplot2 R package (Version 3.3.5) to display the ECM gene set to expression and low expression. R package pheatmap (Version 1.0.12) was used to draw heatmaps to visualise the scaled expression levels of target genes in different cell subsets.

### 5.16 Differential gene expression analysis

We used the Wilcoxon rank test with Seurat functions FindMarkers to identify differentially expressed genes (DEGs) between different segments. DEGs for each cell type were split by up‐ and down‐regulated expression. Due to the higher expression of ECM‐encoding genes in aganglionic segments, we focused on up‐regulated DEGs at this time. scRNA‐seq data are split by subject, and the ECM‐related cellular subset is subsequently extracted.

For HSCR segments, the normal segment of each subject serves as his or her own control of the aganglionic segment, allowing sufficient power to detect a significant effect of the segment with only a small number of subjects. The genes that appeared in the three or above subjects were the potential DEGs. The number of myofibroblasts and smooth muscle cells was too few to perform the differential expression analysis in several subjects, so we merged the two cell types for further analysis.

Since myofibroblasts and smooth muscle cells are close to each other in UMAP cluster visualisation, their gene expression pattern and state are similar. There is a biological transformation relationship between myofibroblasts and smooth muscle cells, so we combined the two cell types for further analysis. The number of myofibroblasts in several samples is too small to perform the differential analysis, and samples would also need to be combined to facilitate differential analysis.

The intersection of different comparisons was visualised using the UpSetR package (Version 1.4.0). Scaled gene expression levels between groups were visualised using pheatmap (Version 1.0.12). In addition, to explore the expression alterations of interesting genes among different segments in ECM‐related subsets, the Kruskal–Wallis test and the Wilcoxon rank‐sum test were performed and visualised as violin plots through ggpubr (Version 0.4.0) and the ggplot2 R package (Version 3.3.5).

### 5.17 Gene ontology enrichment analysis

GO enrichment analyses were performed for the target DEGs with the clusterProfiler R package^94^ function enrichGO (*p*‐value cutoff = .05, *p* adjust method = ‘BH’, *q*‐value cutoff = .1).

To focus on the alteration in the aganglionic segment, the up‐regulated DEGs in the intersection (aganglionic vs. normal, aganglionic segments vs. distal healthy control segments) of ECM‐related cellular subsets were chosen as the target DEGs. Significantly enriched GO terms in the biological processes category were included for word cloud annotation attached to the heatmap, which shows the general biological functions of the GO terms in each cluster using the simplifyEnrichment (Version 1.7.1) R package.

Significantly enriched GO terms that were associated with HSCR phenotypes (e.g., regulation of ECM, neural development, cell migration, regulation of apoptosis) were visualised using a barplot with the ggplot2 R package.

### 5.18 Single‐cell trajectories construction

To explore the activation of myofibroblasts in different intestinal segments, the single‐cell trajectories among segments were constructed to reshape the changes of myofibroblasts over time. Monocle R package (Version 2.18.0)^95^ was used for single‐cell trajectory analysis, and the ggplot2 R package (Version 3.3.5) was used to visualise the results of single‐cell trajectory.

Myofibroblast and its original cell type (fibroblasts, pericytes) were extracted and split into different segments. Each segment data were used to build the single‐cell trajectory, and the fibroblast was chosen as the beginning of the trajectory according to the known biological process of myofibroblast differentiation. To further analyse the trajectories of myofibroblast activation among intestinal segments, we observe the changes in the expression levels of the myofibroblast marker *ACTA2* and *ACTG2*, fibrosis‐related growth factor *POSTN* and the myofibroblast regulatory factor *TGFB1*.

### 5.19 Transcription factors inference

This study performed single‐cell regulatory network inference and clustering (SCENIC) to infer transcription factors associated with abnormal ECM expression at the single‐cell level.^96^

ECM‐related cellular subsets were extracted and converted to .h5ad format using SeuratDisk (Version 0.0.0.9020) R package, and the transcription factor list allTFs_hg38.txt and species‐specific dataset (https://resources.aertslab.org/cistarget/) were also prepared. Then, we read and analysed each dataset using pySCENIC (Version 0.11.2) according to the online tutorials (https://pyscenic.readthedocs.io/en/latest/tutorial.html).

We selected the top 10 regulons from each segment using an RSS panel plot. The method for visualising expression levels of transcription factors is the same as described above. Moreover, we performed the corrplot to visualise the correlation matrix involved in the expression levels of the top 10 regulons and HSCR‐related ECM genes using the corrplot R package (Version 0.92).

### 5.20 Statistical analysis

For scRNA‐seq analysis, we used Wilcoxon rank test with Seurat R package functions FindMarkers (adjusted *p* < .05, |Log2FC| > 0.25) to identify DEGs. ECM‐related cellular subsets were extracted and analysed in two comparisons (aganglionic segments vs. distal healthy control segments; normal segments vs. proximal healthy control segments). For bulk transcriptomics analysis, the genes with absolute log‐fold‐change greater than 2 and *p*‐value less than .05 were included to validate the scRNA‐seq results. For proteomics analysis, the detailed analysis process can be found in the original study. For RT‐qPCR validation, *p*‐value was calculated from Wilcoxon matched‐pairs signed‐rank test in three comparisons (aganglionic segments vs. normal segments, transition segments vs. normal segments and aganglionic segments vs. transition segments) per subject.

## CONFLICT OF INTERESTS STATEMENT

The authors declare no conflicts of interest in this work.

## Supporting information

Supporting Information

Supporting Information

Supporting Information

Supporting Information

Supporting Information

Supporting Information

Supporting Information

Supporting Information

Supporting Information

Supporting Information
