# Supplementary figures and images for: Intestinal fibrosis in aganglionic segment of Hirschsprung's disease revealed by single‐cell RNA sequencing
Source: Clin Transl Med. 2023 Feb 3;13(2):e1193. doi: 10.1002/ctm2.1193 (PMC9898741; doi:10.1002/ctm2.1193)

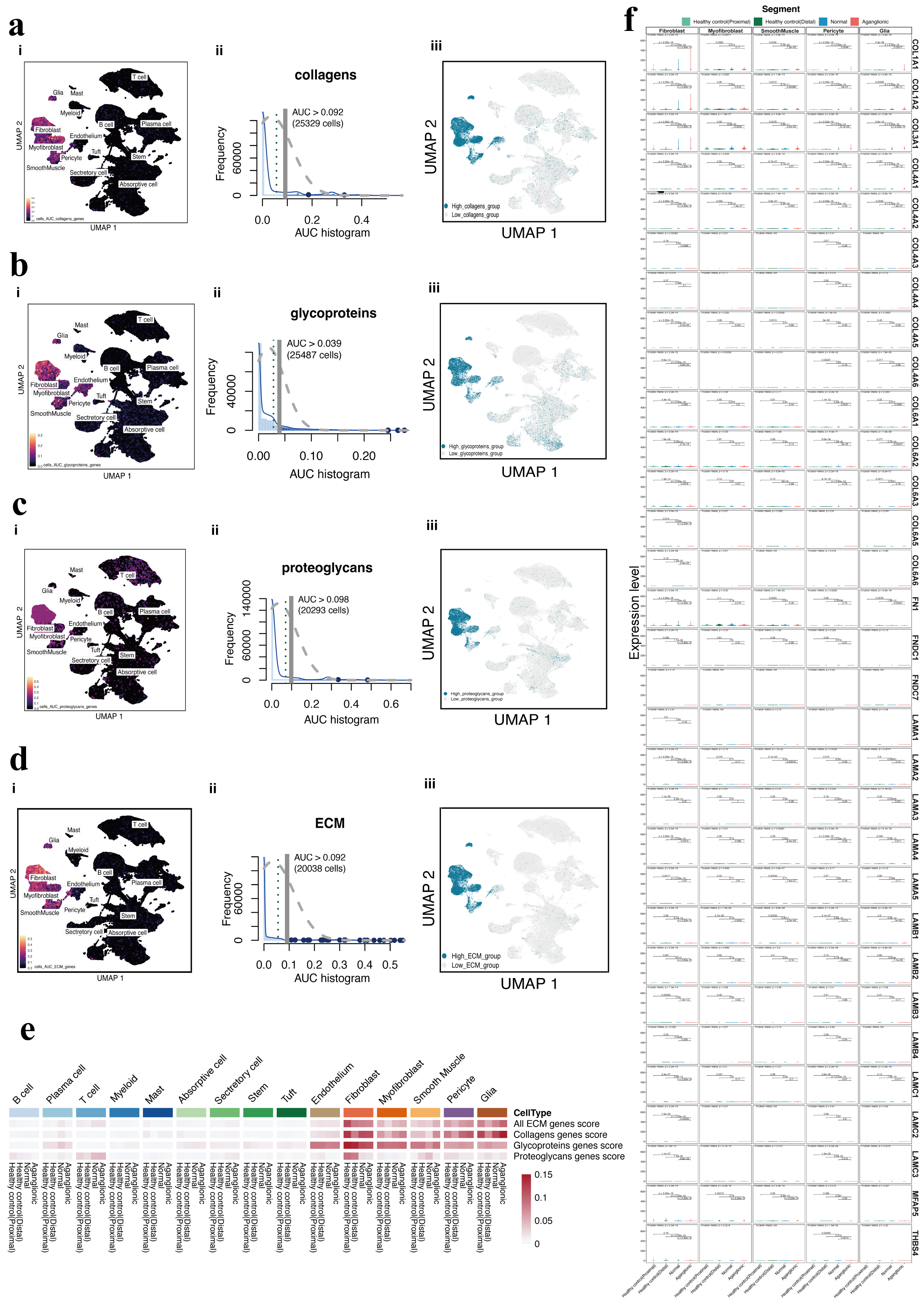

Supplement: Supplementary file 1 — Supporting Information [file CTM2-13-e1193-s007.tif]

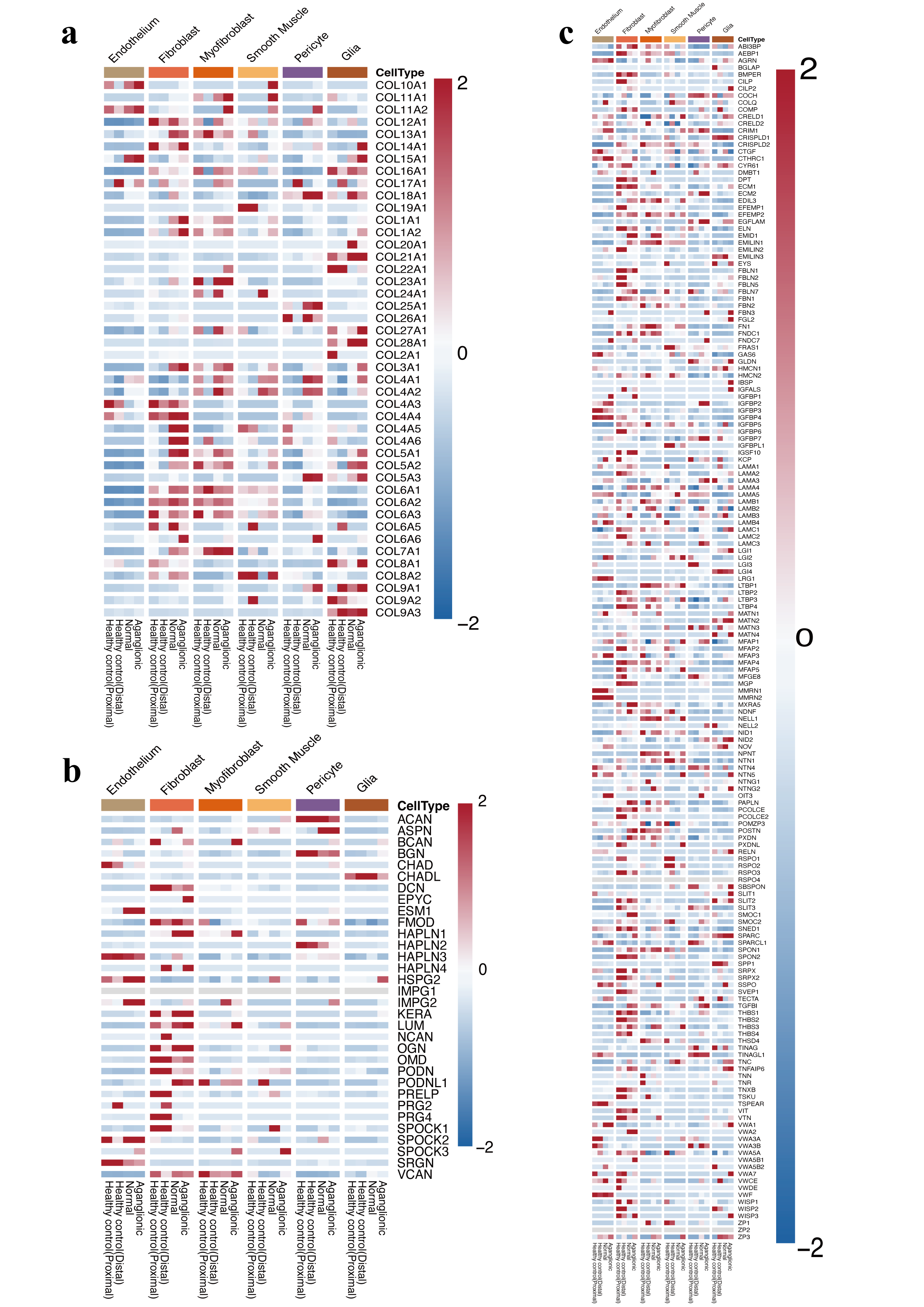

Supplement: Supplementary file 2 — Supporting Information [file CTM2-13-e1193-s008.tif]

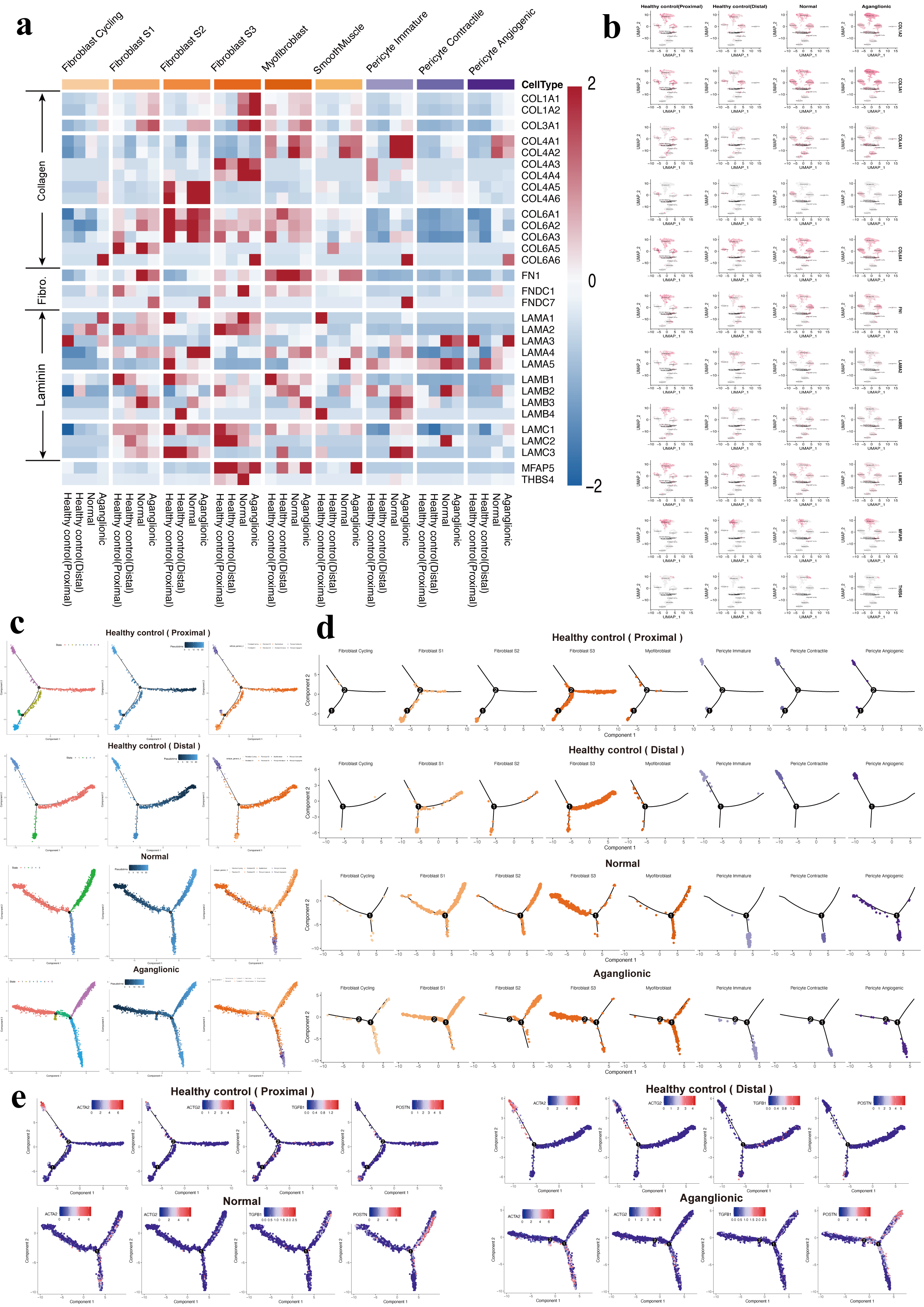

Supplement: Supplementary file 3 — Supporting Information [file CTM2-13-e1193-s004.tif]

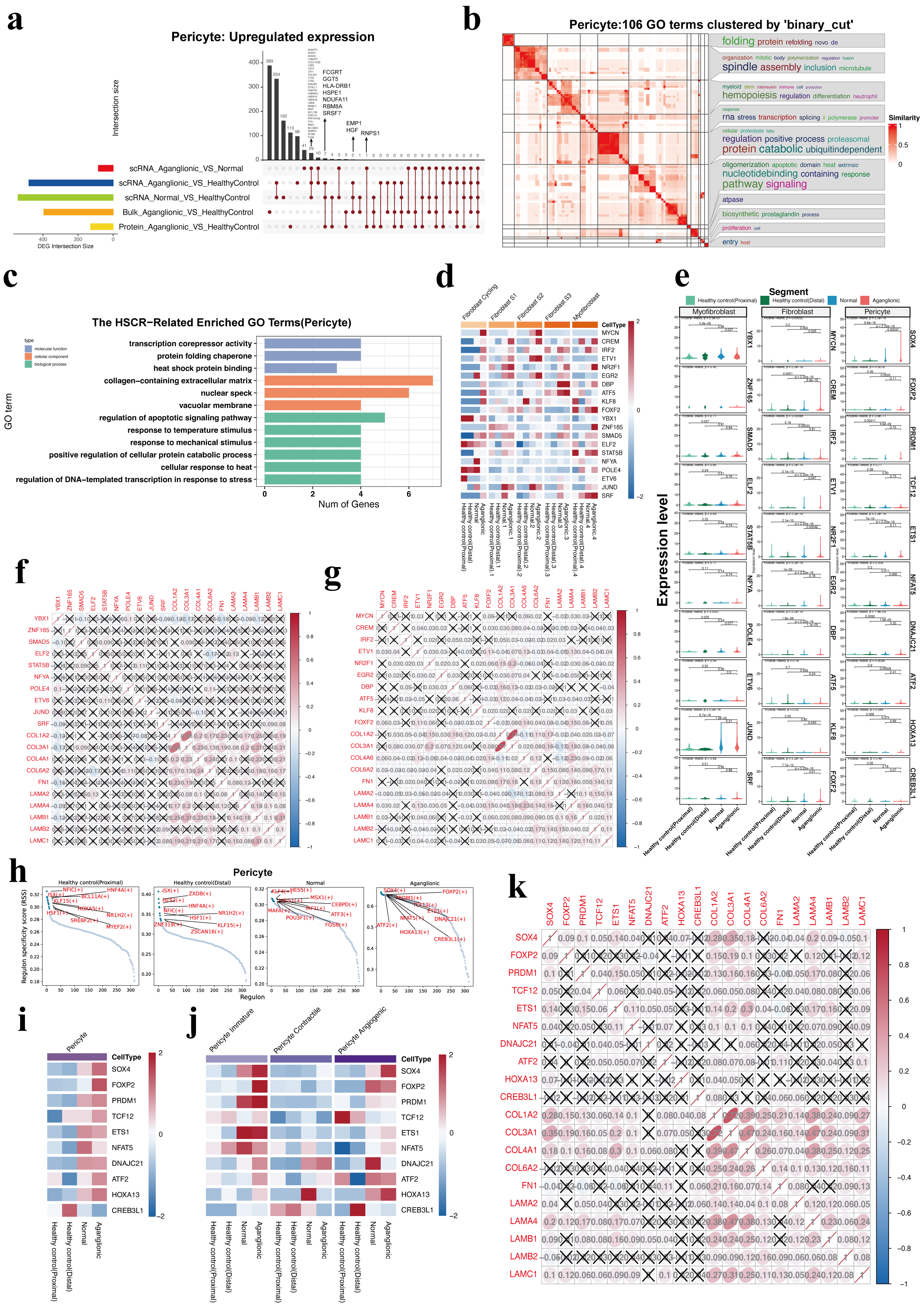

Supplement: Supplementary file 4 — Supporting Information [file CTM2-13-e1193-s005.tif]

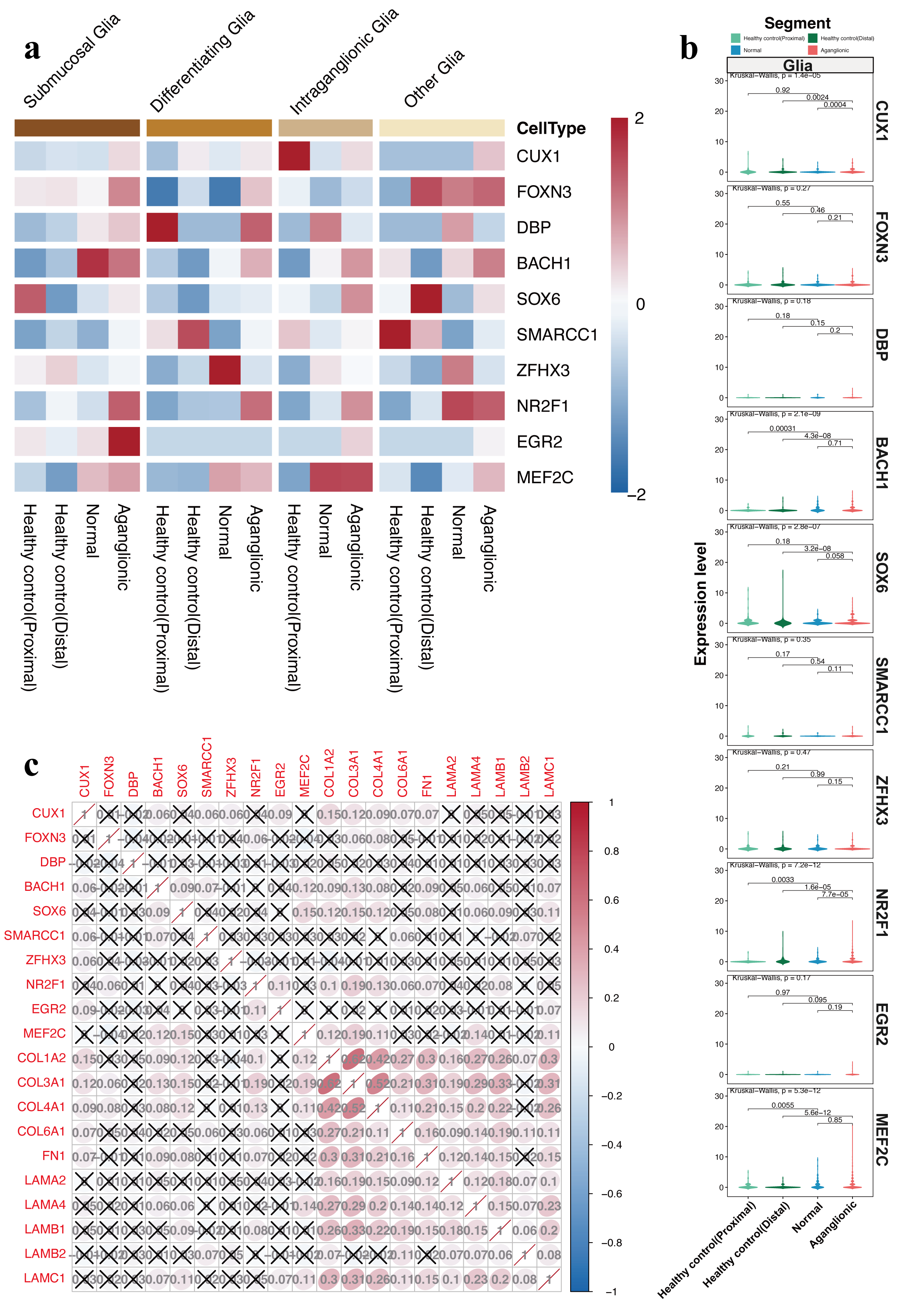

Supplement: Supplementary file 5 — Supporting Information [file CTM2-13-e1193-s010.tif]
