# Supplementary material for: Intestinal fibrosis in aganglionic segment of Hirschsprung's disease revealed by single‐cell RNA sequencing
Source: Clin Transl Med. 2023 Feb 3;13(2):e1193. doi: 10.1002/ctm2.1193 (PMC9898741; doi:10.1002/ctm2.1193)
Supplement: Supplementary file 10 — Supporting Information [file CTM2-13-e1193-s003.docx]

**Supplementary Figure 1. Source of extracellular matrix in the intestine**

1. UMAP is colored based on the AUC scores of collagens gene set (i). The AUC histogram of collagens gene set, the grey line is the threshold suggested for the collagens gene-set (ii).To highlight the cluster of cells that are more likely of the cell type according to the signatures, we split the cells into the cells that pass the assignment threshold (colored in shades of blue), and the cells that don’t (colored in light grey) in the UMAP (iii).
2. UMAP is colored based on the AUC scores of glycoproteins gene set (i). The AUC histogram of glycoproteins gene set, the grey line is the threshold suggested for the glycoproteins gene-set (ii).To highlight the cluster of cells that are more likely of the cell type according to the signatures, we split the cells into the cells that pass the assignment threshold (colored in shades of blue), and the cells that don’t (colored in light grey) in the UMAP (iii).

(c)UMAP is colored based on the AUC scores of proteoglycans gene set (i). The AUC histogram of proteoglycans gene set, the grey line is the threshold suggested for the collagens gene-set (ii).To highlight the cluster of cells that are more likely of the cell type according to the signatures, we split the cells into the cells that pass the assignment threshold (colored in shades of blue), and the cells that don’t (colored in light grey) in the UMAP (iii).

(d)UMAP is colored based on the AUC scores of all ECM gene set (i). The AUC histogram of all ECM gene set, the grey line is the threshold suggested for the all ECM gene-set (ii).To highlight the cluster of cells that are more likely of the cell type according to the signatures, we split the cells into the cells that pass the assignment threshold (colored in shades of blue), and the cells that don’t (colored in light grey) in the UMAP (iii).

(e) Heatmap of AUC scores of collagens, glycoproteins, proteoglycans, and all ECM gene set grouped by segments. The color bars match the cell-type group colors.

(f) Violin plot of HSCR-related ECM genes among different segments in merge data set. The results of differential analysis are mapped on the Violin plot. Kruskal-Wallis Test was used in multi-segments differenial analysis, and wilcox test was used in the differenial analysis between groups.

**Supplementary Figure 2. Heatmap of the ECM gene set in ECM-related subset**

(a) Heatmap of collagens genes among different segments in ECM-related subset. The color bars match the cell-type group colors.

(b) Heatmap of proteoglycans genes among different segments in ECM-related subset. The color bars match the cell-type group colors.

(c) Heatmap of glycoproteins genes among different segments in ECM-related subset. The color bars match the cell-type group colors.

**Supplementary Figure 3. Heterogeneity of stromal cell and origin of myofibroblasts**

1. Heatmap of HSCR-related ECM genes among different segments in stromal subset. The color bars match the cell-type group colors.
2. Feature plot of HSCR-related ECM genes among different segments in stromal subset. The color bars match the cell-type group colors.
3. Trajectory plot display trajectory grouped by healthy control, normal, aganglionic segments.
4. Trajectory plot display trajectory splited by different celltype.
5. Trajectory plot display the expression levels of myofibroblast marker ACTA2 & ACTG2, fibrosis-associated myofibroblast markers POSTN, myofibroblast activation regulator TGFB1 along the trajectory.

**Supplementary Figure 4. Heterogeneity of ECM-related stromal cell**

1. Static UpSet plot of the up-regulated DEGs among different set intersections in the pericytes subset.
2. Heatmap of simplying the GO enrichment results in the pericytes subset by clustering the significant GO terms, on the right side of the heatmap there are the word cloud annotations which summarize the functions with keywords in every GO cluster.
3. Barplot of HSCR-related significantly GO terms in the pericytes subset.
4. Heatmap of top 10 regulons among different segments in myofibroblasts and smooth muscle cells and fibroblasts detailed subset. The color bars match the cell-type group colors.
5. Violin plot of top 10 regulon among different segments in ECM-related stromal subsets, and the results of differential analyses.
6. Corrplot of a correlation matrix involved in the expression levels of top 10 regulon and HSCR-realted ECM genes in the myofibroblasts and smooth muscle cells subset.
7. Corrplot of a correlation matrix involved in the expression levels of top 10 regulon and HSCR-realted ECM genes in the fibroblast subset.
8. RSS panel plot of top 10 regulons with all segments in the pericytes subset.
9. Heatmap of top 10 regulons among different segments in pericytes subset. The color bars match the cell-type group colors.
10. Heatmap of top 10 regulons among different segments in pericytes detailed subset. The color bars match the cell-type group colors
11. Corrplot of a correlation matrix involved in the expression levels of top 10 regulon and HSCR-realted ECM genes in the pericytes subset.

**Supplementary Figure 5. Heterogeneity of glial cell**

1. Heatmap of top 10 regulons among different segments in glials detailed subset. The color bars match the cell-type group colors.
2. Corrplot of a correlation matrix involved in the expression levels of top 10 regulon and HSCR-realted ECM genes in the glials subset.
3. Violin plot of top 10 regulon among different segments in glials subsets, and the results of differential analyses.

**Appendices**

Supplementary Figure 1. Source of extracellular matrix in the intestine

Supplementary Figure 2. Heatmap of the ECM gene set in ECM-related subset

Supplementary Figure 3. Heterogeneity of stromal cell and origin of myofibroblasts

Supplementary Figure 4. Heterogeneity of ECM-related stromal cell

Supplementary Figure 5. Heterogeneity of glial cell

Supplementary Table 1. The detailed information of subjects in scRNA-seq and RT-qPCR

Supplementary Table 2. The cell markers used in scRNA-seq

Supplementary Table 3. The detailed information of all primers used in RT-qPCR

Supplementary Table 4. The differential expression genes of all cell types
